# One in five women suffer from pelvic floor disorders in Kersa district Eastern Ethiopia: a community-based study

**DOI:** 10.1186/s12905-018-0585-1

**Published:** 2018-06-15

**Authors:** Merga Dheresa, Alemayehu Worku, Lemessa Oljira, Bizatu Mengiste, Nega Assefa, Yemane Berhane

**Affiliations:** 1 College of Health and Medical Sciences, Haramaya University, Harar, Ethiopia; 2 Addis Continental Institute of Public Health, Addis Ababa, Ethiopia; 3 School of Public Health, Addis Ababa University, Addis Ababa, Ethiopia; 4 Harar, Ethiopia; 5 Addis Ababa, Ethiopia

**Keywords:** Pelvic Floor Disorder, Women, OAB, SUI, POP, AI, Kersa, Ethiopia

## Abstract

**Background:**

Hundreds of millions of women suffer from pelvic floor disorders globally, often in silence. Women in developing countries do not disclose their problems due to associated social stigma or lack of access to services. Thus, the extent of the problem remains largely unknown. This study was conducted to assess the magnitude of pelvic floor disorders in Kersa district Eastern Ethiopia.

**Method:**

We conducted a community-based cross-sectional study among ever married women who reside in Kersa district, Eastern Ethiopia. The study subjects were selected through stratified multistage probability sampling. The data were collected using a structured questionnaire through face-to-face interviews. The prevalence of various pelvic floor disorders are presented along with the 95% Confidence Intervals (CI).

**Results:**

A total of 3432 women participated in the study, of which 704 (20.5%; 95% CI; 19.2, 21.8) reported at least one type of pelvic floor disorder and 349 (49.6%; 95% CI: 46.0, 53.0) reported two or more pelvic floor disorders. The most common pelvic floor disorders included an over active bladder (15.5%; 95% CI: 14.4, 16.8), pelvic organ prolapse (9.5%; 95% CI: 8.5,10.4), stress urinary incontinence (8.3%; 95% CI: 7.4, 9.2) and anal incontinence (1.9%; 95% CI: 1.5, 2.4). More than two-thirds of the women with pelvic floor disorders (68.0%; 95% CI:64.4, 71.3) reported having severe distress but had never sought health care.

**Conclusions:**

The magnitude of the health problem and the low level of health seeking behavior indicates the silent suffering of many women in the study area. Extrapolating these figure to national statistics would indicate the staggering number of women suffering from pelvic floor disorders in the country. This calls for urgent action to improve prevention, diagnosis and treatment services to mitigate the suffering of women from pelvic floor disorders.

## Background

Pelvic floor disorders (PFDs) affect hundreds of millions of women globally [1, 2]. PFDs cause serious social, economic, physical and psychological problems for women that significantly reduce their quality of life and productivity [3, 4]. Studies have shown that 11%-35.5% of women suffer from PFDs globally [5, 6]. The most common PFDs include over active bladder (OAB) with an estimated prevalence of 7.6%-13% [7, 8], stress urinary incontinence (SUI) with an estimated prevalence of 1.7% - 35.5%, [9, 10], pelvic organ prolapse (POP) with an estimated prevalence of 2.9%-20% [9, 11] and anal incontinence (AI) with an estimated prevalence of 0.2 - 13% [5, 12].

PFDs are usually associated with a high number of pregnancies and deliveries [13, 14] and heavy lifting [5, 15]. The tendency to develop PFDs increases with age due to the weakening of pelvic floor muscles [1] mostly after the age of 55 years [2, 16]. Although PFDs are distressing and cause serious discomfort, women in developing countries often endure the pain and discomfort in silence [17, 18] and many do not seek health care for the problem [19].

Despite widespread recognition of the problem among maternal health experts, studies to assess the magnitude of pelvic floor disorder are scarce in developing countries [5, 18]. This is the first large scale community-based study to assess the magnitude of PFDs in Ethiopia.

## Methods

### Study setting and design

A community-based cross-sectional study was conducted in Kersa district, East Hararghe Zone, Ethiopia. The study population was ever married women residing in Kersa Health and Demography Surveillance System (Kersa HDSS) field site. The study was conducted from August 10 to September 4, 2016. The study district has 38 *kebeles* (smallest administrative unit in Ethiopia) of which 24 randomly selected *kebeles* were included in Kersa HDSS. The district has 6 health centers and 20 health posts. The site constitutes 26,061 household and 127,000 inhabitants of which 23% are women of reproductive age [20].

### Population and sampling technique

The study population were ever married women who had resided in Kersa HDSS field site for at least six months. For interviewing unmarried women about their gynecological morbidity is a taboo in traditional community, unmarried women were excluded from this study[21, 22]. A multi-stage, stratified, random sampling procedure proportional to the size of the household in each of the 24 *kebeles* was used to enroll the study participants. The allocated sample size to each *kebeles* was further assigned to each *ketena* (*subunit of kebeles*) proportional to the size of the household. Sample size was determined for a larger study on identifying factors associated with PFD. With an assumption of 95% CI, power 80, and prevalence of PFD among nulliparous women of 13% and of parous at 18% [23], and a design effect of two, a sample size of 3444 was calculated. The adequacy of the sample to determine the magnitude of PFD was checked. Kersa HDSS database was used as a sampling frame: the name of the selected women and their household ID was retrieved from the database and printed in hardcopy for use in the field.

### Data collection tools

A structured data collection tool was customized and adapted from the literature [12, 24, 25]. The questionnaire contained questions about socio-demographic factors, obstetric history and pelvic floor disorder symptoms. Initially, the content of the questionnaire was validated by three gynecologists and two reproductive health experts. The original questionnaire was prepared in English language and later translated into the local languages (Amharic and Afan Oromo) for data collection. Forward and backward translations were performed by two bilingual translators. We pretested the questionnaire in a similar setting and refined the tool based on the feedback we obtained. The questionnaire had good internal consistency (Cronbach alpha ranges from 0.82 to 0.89).

Experienced female data collectors and field supervisors who can speak the local languages fluently were recruited and trained for four days. The training for field workers included field procedures, interviewing techniques and discussion on the content of the data collection tool. A field guide and data collection manual were used as a reference during the training. Field supervisors checked compliance with field procedures and the completeness of questionnaires in the field.

### Measurement

Pelvic floor disorder was assessed based on women’s reporting of symptoms. Each pelvic floor disorder (Stress urinary incontinences (SUI), over active bladder (OAB), pelvic organ prolapse (POP), and anal incontinence (AI)) was dichotomized as present or absent based on responses to each symptom domain. Over active bladder was assessed by asking, “Do you rush to urinate so that you will not have leakage of urine?”, “Do you experience frequent urination?”, “Have you experienced urine leakage related to a feeling of urgency?” Stress urinary incontinence was assessed by asking, “Do you experience urine leakage related to activity, coughing, or sneezing?” and “Do you experience small amounts of urine leakage (drops)?” Pelvic organ prolapse was assessed by asking, “Do you have a sensation that there is a bulge in your vagina or that something is falling out from your vagina?” Anal incontinence was assessed by asking, “Do you lose gas from your rectum that is beyond your control?”, “Do you lose stool beyond your control if your stool is loose or liquid?”, “Do you lose well-formed stool beyond your control?” Positive responses to at least one of the questions from each of the pelvic floor disorder categories defined the presence of the problem. Women who had at least one pelvic floor disorder were categorized as “have PFD” and women who did not report at least one pelvic floor disorder were categorized as “do not have PFD” [12, 24, 25].

To assess the degree of distress caused by the symptoms each PFD symptom question was assessed by a four-point Likert scale. If symptoms were present the individual was asked “How much are you bothered by the symptoms?” and the response was rated from ‘not at all=1’ to ‘quite a bit=4’. The mean value of symptom distress was multiplied by 25 to obtain the score ranges from 0-100 for each domain: bladder symptoms (both OAB and SUI), AI, and POP[26]. Severity of the symptoms among women with PFD was determined based on distress score ranges from 1-100 and categorized by tertiles(in to three part) as ‘mild symptoms’ if the total score was 1-33, ‘moderate’ if the score was 34-66, and ‘severe’ if the score was 67-100[27].

### Data analysis

Data were double entered into Epi-Data 3.1 to check consistency and exported to STATA version 14 for analysis. The prevalence of each PFD was calculated by dividing the number of women who reported the symptoms by the total number of women in the study and was reported with 95% Confidence Intervals (CI). The overall prevalence of PFD was calculated by dividing the number of women who reported at least one of the symptoms of PFD by total number of women in the study and reported with a 95% CI. The severity of distress caused by the symptoms was described as mild(1-33), moderate(34-66) and severe(37-100) and presented as a proportion with 95% CI.

## Results

A total of 3432 ever married women participated in the study which resulted in a 99.6 % response rate. Their age ranged from 15 to 80 years with the mean (standard deviation (SD)) of 36.5 (+13) years. The mean (SD) age at first marriage was 16.2 (+1.8) years and the mean (SD) age at first child birth was 18.0 (+1.8) years. Seventy-nine percent (2724) of the women had not attended school and 391(12.0 %) of them had married more than once (Table 1).

**Table 1:** Socio-demographic characteristics of study participants living in Kersa HDSS, Ethiopia 2016

Variable		Number	Percent
Age of respondents (*n*=3432)	15-24 year	509	14.8
	25-34 year	1202	35.0
	35-44 year	825	24.0
	45-54 year	420	12.2
	55+	476	14.0
Educational status (*n*=3432)	No schooling	2724	79.0
	Has some education	708	21.0
Occupation(*n*=3432)	House wife	3202	93.0
	Self-employee	155	4.5
	Government Employee	16	0.5
	Unemployed	59	2.0
Marital status(*n*=3432)	Currently Married	2,922	85.0
	Widowed/Divorced	510	15.0
Numbers of married times (*n*=3373)	Once	2982	88.0
	More than once	391	12.0
Age at first marriage (*n*=3432)	<18 years old	2673	78.0
	>=18 years old	759	22.0

Two thousand four hundred sixty-five (74.3%) of the women had never given birth at a health facility. The mean number of pregnancies per woman was 5.9 (with a range from 1 to 17) and the mean number of deliveries was 5.6 (with a range from 1 to 16). About one-in-six (16.0%) of the women had ever had an abortion and only 38 (1.0%) had ever had a cesarean delivery (Table 2).

**Table 2:** Reproductive health history of study participants living in Kersa HDSS, Ethiopia, 2016

Variable		frequency	Percent
Ever had pregnancy (*n*=3432)	Yes	3337	97.0
	No	95	3.0
Number of pregnancies (*n*=3337)	4 and less	1260	38.0
	5 and above	2077	62.0
History of abortion (*n*=3337)	Yes	535	16.0
	No	2802	84.0
Ever had child birth (*n*=3337)	Yes	3317	99.0
	No	20	1.0
Number of child birth (*n*=3317)	1-4	1326	40.0
	5 and above	1991	60.0
Mode of delivery at first child birth (*n*=3317)	vaginal	3294	99.0
	cesarean section	23	1.0
Ever had vaginal delivery (*n*=3317)	Yes	3309	99.8
	No	8	0.2
Number of vaginal deliveries (*n*=3317)	4 and less	1319	40.0
	5 and above	1998	60.0
Home delivery (*n*=3317)	Never at home	226	7.0
	Ever at home	3091	93.0
Institution delivery (*n*=3317)	Never at health facility	2465	74.0
	Ever at health facility	852	26.0
Episiotomy during delivery (*n*=3255)	Yes	331	10.0
	No	2924	90.0
Ever cesarean delivery (*n*=3302)	Yes	38	1.0
	No	3264	99.0
Frequency of cesarean section (*n*=38)	One time	28	74.0
	More than one time	10	26.0
Menopause(*n*=3432)	Yes	859	25.0
	No	2573	75.0

Overall, 704 (20.5%) [95% (CI): 19.2, 21.8] of the women reported at least one type of pelvic floor disorder. The magnitudes of each pelvic floor disorder with their 95 % CI were 15.5% [14.4, 16.8] for Over Active Bladder (OAB), 8.3% [7.4, 9.2] for Stress Urinary Incontinence (SUI), 9.5% [8.5, 10.4] for Pelvic Organ Prolapsed (POP) and 1.9% [1.5, 2.4] for Anal Incontinence (AI) (Table 3).

**Table 3:** Prevalence and co-occurrence of pelvic floor disorder among study participants living in Kersa HDSS, Ethiopia, 2016

Variable	All women (*N*=3432)	
	Number	%(95% CI
Any Pelvic Floor Disorder(*n*=3432)	**704**	**20.5(19.2, 21.8)**
Only one disorder(*n*=704)	355	50.4(46.7, 54.0)
Two or more disorders(*n*=704)	349	49.6(46.0,53.0)
Two disorders (*n*=704)	219	31.1(27.7, 34.6)
Three disorders(*n*=704)	102	14.5(12.0, 17.0)
All four disorders (*n*=704)	28	3.9(2.7, 5.7)
Over Active Bladder (OAB)(*n*=704)	**534**	**15.5(14.4-16.8)**
OAB only(*n*=534)	195	36.5(32.5, 40.7)
OAB with SUI(*n*=534)	264	49.4(45.0, 53.6)
OAB with AI(*n*=534)	48	9(8.5, 10.4)
OAB with any other PFD(*n*=534)	339	63.5(59.0, 67.0)
Stress Urinary Incontinence (SUI)(*n*=704)	**285**	**8.3(7.4, 9.2)**
SUI only(*n*=285)	12	4.2(2.3, 7.2)
SUI with AI(*n*=285)	37	12.9(9.5, 17.0)
SUI with any other PFD(*n*=285)	273	96.0(93.0, 97.0)
Pelvic Organ Prolapse (POP)(*n*=704)	**325**	**9.5(8.5, 10.4)**
POP only (*n*=325)	131	40.3(35.0, 46.0)
POP with SUI(*n*=325)	124	38.0(33.0, 43.5)
POP with OAB (*n*=325)	184	56.6(51.0, 62.0)
POP with AI (*n*=325)	36	11.0(8.0, 15.0)
POP with SUI and OAB(*n*=325)	115	35.3(30.3-40.7)
POP with any other PFD(*n*=325)	194	59.6(54.0, 65.0)
Anal Incontinence (AI )(*n*=704)	**67**	**1.9(1.5, 2.4)**
AI Only(*n*=67)	17	25.3(16.0, 37.0)
AI with any other PFD (*n*=67)	50	74.6(62.5, 83.8)

Any= indicate prevalence of participating women with indicated outcome without excluding the existance of other problems. Only=indicates the prevalence of outcome without co ocurance of other outcome

**Fig. 1:**
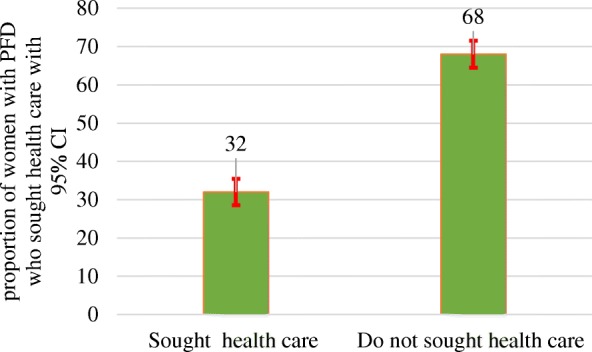
Proportion of women with pelvic floor disorder who sought health care services, living in Kersa HDSS, 2016

The prevalence of pelvic floor disorders was significantly higher for older women and women with high parity (Table 4). From those women who had experienced PFDs, only 32.0% [95% CI: 28.6, 35.5] had sought health care services (Fig. 1).

**Table 4:** Pelvic floor disorders by age and parity of study participants living in Kersa HDSS, Ethiopia, 2016

Variable	OAB n/N=534/3432		SUI n/N= 285/3432		POP n/N=325/3432		AI n/N=67/3432		PFD n/N=704/3432	
	%	percentage (95% Cl)	%	percentage (95% Cl)	%	Percentage (95% Cl)	%	Percent (95% Cl)	%	Percent (95% Cl)
Overall prevalence	15.5	(143.-16.8)	8.3	(7.4-9.2)	9.5	(8.5-10.4)	1.9	(1.5-2.4)	20.5	(19.1-21.8)
Age of respondents										
15-24 year	11.7	(9.2-14.8)	5.3	(3.6-7.6)	4.1	(2.7-6.2)	0.9	(0.4-2.3)	13.9	(11.1-17.2)
25-34 year	11.8	(10.1-13.8)	5.8	(4.6-7.2)	7.9	(6.5-9.6)	1.2	(0.7-2.0)	16.5	(14.5-18.7)
35-44 year	18.1	(15.6-20.9)	10.7	(8.8-13.0)	12.4	(10.3-14.9)	2.6	(1.6-4.0)	24.1	(21.3-27.1)
45-54 year	18.5	(15.5-22.5)	8.5	(6.2-11.6)	11.4	(8.7-14.8)	3.3	(1.9-5.5)	25.0	(21.0-29.3)
55+ year	21.6	(18.1-25.5)	13.2	(10.4-16.5)	11.9	(9.3-15.2)	2.3	(1.2-4.1)	27.3	(23.4-31.4)
Parity										
1-4	12.2	(10.5-14.0)	6.1	(5.0-7.6)	7.3	(6.0-8.9)	1.0	(0.6-1.7)	16.8	(14.9-19.0)
=>5	17.9	(16.3-19.6)	10.0	(8.7-11.3)	10.9	(9.5-12.3)	2.5	(1.9-3.2)	23.0	(21.3-25.0)

*OAB* Over Active Bladder, *SUI* Stress Urinary Incontinence, *POP* Pelvic Organ prolapse, *PFD* Pelvic Floor Disorder

**Fig. 2:**
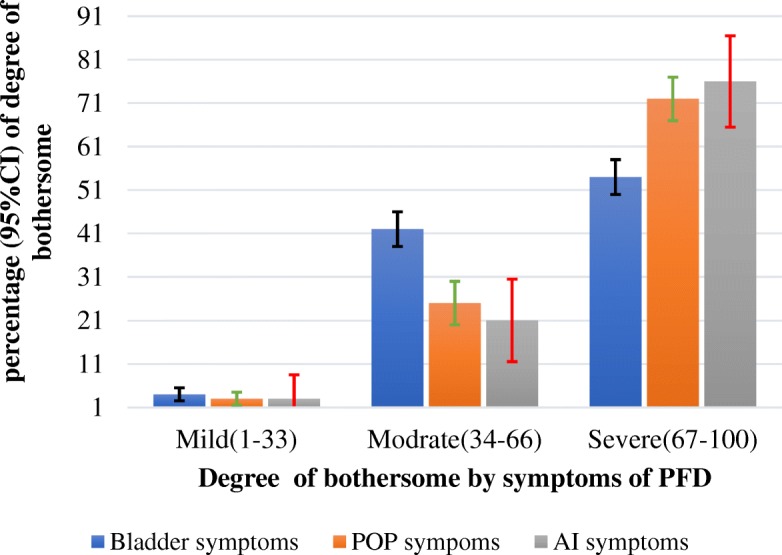
Severity of symptoms among participating women with pelvic floor disorder, living in Kersa HDSS, Ethiopia, 2016

The majority of women with at least one PFD reported being seriously worried about their disorder: 57.0 % of those with urinary symptoms, 72.0 % of women with POP, and 76.0 % of women with AI were severely (67-100) bothered by the symptoms (Fig. 2). The level of distress increased with multiple co-occurrences of the PFDs: 53.0 % of those with one PFD, 61.0 % of those with two PFDs, 72 % of those with three PFDs and 78.0 % of those with four PFDs were severely distressed by the symptoms.

## Discussion

The prevalence of pelvic floor disorder in this study was high (20.5%) among ever-married women. Of the women who experienced the disorder, one in six women reported symptoms of over active bladder, one in twelve women reported symptoms of stress urinary incontinence, one in ten women reported pelvic organ prolapse and one in fifty women reported anal incontinence. These figures show pelvic floor disorders are a significant public health problem and there is a need for urgent action.

The results of this study are consistent with a study done in India which showed a prevalence of 21.0% [28] and in USA with a prevalence of 25.0% [11]. On the other hand another study in Ethiopia reported a prevalence of 12.0% [5] which is much less than the current findings. The difference in the reported prevalence can be explained by the variation in study participants; in the former study 8.0% of the participants had never given birth while in our study those who had never given birth were less than 3.0%. Moreover, in the previous study grand multipara women constituted 45.0% of the sample whereas in our study they constituted 60.0% and both childbirth and the number of births are identified as risk factors for PFD [13]. A reported 35.3% prevalence of PFD from Bangladesh [6] is higher than our estimate of 20.5%. The discrepancy between these two estimates may be related to the difference in study population, where in the former study post-menopause age women constituted 41.0% while it was 25.0% in our study.

PFD affects women’s ability to perform household chores, outdoor activities, heavy work, leisure activities, their sexual life, mental health and sense of overall wellbeing [29]. In this study only a third of the women with PFD sought health care. In Ethiopia, although women regard PFD as unnatural and uncommon, most took no action to improve the situation either due to associated stigma or lack of access to appropriate services [18].

In this study OAB was the most prevalent PFD, followed by POP, SUI, and AI. This pattern of PFD is in agreement with Asian and African studies [5, 6, 10, 30]. In Western countries AI and SUI are the most prevalent PFD, followed by OAB. POP is the least reported PFD [11, 31]. The pattern of PFD varies in relation to exposure of women to attributable risk factors. High fetal birth weight, which is associated with fetal macrosomia, leads to prolonged second stage labors and increases the risk of pelvic nerve and anal sphincter injuries. These injuries are major risk factors for anal incontinence [32]. In developing countries, where the prevalence of low birth weight is high [33], macrosomia leading to obstetric anal sphincter injury is less common.

Furthermore, UI and AI prevalence varies with biological or racial difference. Higher prevalence of OAB and lower prevalence of UI has been observed among black women as compared to white women [2]. The difference in pattern is explained by a higher urethral closure pressure in black women during a maximum pelvic muscle contraction than other races [34]. Vaginal delivery and multiparity, which lead to levatorani muscle avulsion, over distention, reduction in muscle strength and increased hiatal area are the main risk factors for POP [12]. In Ethiopia vaginal delivery is practiced by almost all women (Table 2), the total fertility rate is 4.6 [35] and women also commonly engage in manual work even while pregnant or shortly after delivery [36]. These factors contribute to high rates of pelvic organ prolapse.

Women feel shame and are embarrassed about their PFD and suffer in silence. Creating awareness will help women to discuss PFD with their partners and care providers [37]. Disclosing their condition to others may help them to get advice about treatment. Women with POP have difficulties with sexual intimacy due to the sensation of the prolapse [38], which then affects their reproductive capacity and relationship with their partner.

Urinary and anal incontinence are socially debilitating problems which cause severe emotional distress. Rural women cannot manage incontinence directly because of a lack of underwear, disposable pads and limited access to washing facilities, causing problems with cleanliness and odor. This condition can lead to social isolation, embarrassment, loss of employment, failure to engage in daily activities and impacts on personal and intimate relationships, sometimes leading to divorce and isolation [18].

Co-occurring symptoms of PFD were increased twofold among women who had a vaginal delivery. Co-occurrence of all types of PFDs are strongly related to subsequent vaginal deliveries [12, 32]. In developing countries where the total fertility rate is high and vaginal delivery is almost universal, frequent co-occurrence of PFD is expected. Notably, high co-occurrence of PFD implies a multifaceted burden of disease for women in developing countries.

Women with any type of PFD (OAB, SUI, POP, and AI) symptoms are distressed by their symptoms. More than half of the women with urinary symptoms and three quarters of the women with POP and AI were severely distressed by their symptoms. Severity of distress worsened with the number of co-existing PFDs [12]. Previous studies in Ethiopia revealed that 67.7% of women with PFD had symptoms of depression [17]. Women who live with PFD, suffer from social isolation and face difficulties, both as a woman and a wife [18]. PFD affects the social and family role of women in the community: the symptoms cause women to be confined to the home and lose their mobility, unable to engage in any social activities outside of the home.

The Ethiopian Demographic and Health Survey (EDHS) and other national health surveys do not assess the full range of gynecological morbidities. However, the latest EDHS included questions about obstetric fistula and reported a prevalence which is less than 1%. [35] Thus, pelvic floor disorders, which affect one in five women as reported in this study, never received attention at the level of policy.

The strengths of this study include the inclusion of a large representative sample size, being community-based, obtaining a high response rate, using a validated data collection tool and using female data collectors. Thus, the findings provide a reasonable estimate of the magnitude of PFDs and can be generalized to a large segment of the rural agrarian women in Ethiopia. However, we have not done pelvic examinations, since that would be unacceptable during a household survey, and it is possible that mild cases have been missed due to under reporting [5]. In addition, women may have under reported symptoms due to social stigma and shame [18, 37]. Thus, the prevalence of PFD reported in this study could be an underestimate of the true magnitude.

## Conclusion

In conclusion, PFDs are a major public health problem for women and the majority of women suffering from PFDs do not access appropriate health care. This is a call for urgent action in order to initiate and strengthen prevention, diagnosis, and treatment services for PFDs.

## Abbreviations

AI: Anal Incontinence
CI: Confidence Interval
HDSS: Health Demography Surveillance System
OAB: Over Active Bladder
PFD: Pelvic Floor Disorder
POP: Pelvic Organ Prolapse
SUI: Stress Urinary Incontinence
